# Dichloroacetate Affects Mitochondrial Function and Stemness-Associated Properties in Pancreatic Cancer Cell Lines

**DOI:** 10.3390/cells8050478

**Published:** 2019-05-18

**Authors:** Tiziana Tataranni, Francesca Agriesti, Consiglia Pacelli, Vitalba Ruggieri, Ilaria Laurenzana, Carmela Mazzoccoli, Gerardo Della Sala, Concetta Panebianco, Valerio Pazienza, Nazzareno Capitanio, Claudia Piccoli

**Affiliations:** 1 Laboratory of Pre-Clinical and Translational Research, IRCCS-CROB, Referral Cancer Center of Basilicata, 85028 Rionero in Vulture (Pz), Italy; tiziana.tataranni@crob.it (T.T.); francesca.agriesti@crob.it (F.A.); vitalba.ruggieri@crob.it (V.R.); ilaria.laurenzana@crob.it (I.L.); carmela.mazzoccoli@crob.it (C.M.); gerardo.dellasala@crob.it (G.D.S.); 2 Department of Clinical and Experimental Medicine, University of Foggia, 71100 Foggia, Italy; consiglia.pacelli@unifg.it (C.P.); nazzareno.capitanio@unifg.it (N.C.); 3 Division of Gastroenterology, IRCCS “Casa Sollievo della Sofferenza” Hospital, 71013 San Giovanni Rotondo, Italy; panebianco.c@gmail.com (C.P.); pazienza_valerio@yahoo.it (V.P.)

**Keywords:** metabolism, mitochondria, cancer stem cells

## Abstract

Targeting metabolism represents a possible successful approach to treat cancer. Dichloroacetate (DCA) is a drug known to divert metabolism from anaerobic glycolysis to mitochondrial oxidative phosphorylation by stimulation of PDH. In this study, we investigated the response of two pancreatic cancer cell lines to DCA, in two-dimensional and three-dimension cell cultures, as well as in a mouse model. PANC-1 and BXPC-3 treated with DCA showed a marked decrease in cell proliferation and migration which did not correlate with enhanced apoptosis indicating a cytostatic rather than a cytotoxic effect. Despite PDH activation, DCA treatment resulted in reduced mitochondrial oxygen consumption without affecting glycolysis. Moreover, DCA caused enhancement of ROS production, mtDNA, and of the mitophagy-marker LC3B-II in both cell lines but reduced mitochondrial fusion markers only in BXPC-3. Notably, DCA downregulated the expression of the cancer stem cells markers CD24/CD44/EPCAM only in PANC-1 but inhibited spheroid formation/viability in both cell lines. In a xenograft pancreatic cancer mouse-model DCA treatment resulted in retarding cancer progression. Collectively, our results clearly indicate that the efficacy of DCA in inhibiting cancer growth mechanistically depends on the cell phenotype and on multiple off-target pathways. In this context, the novelty that DCA might affect the cancer stem cell compartment is therapeutically relevant.

## 1. Introduction

Pancreatic ductal adenocarcinoma (PDAC) is a very aggressive cancer, with a low percentage of affected patients eligible for surgical resection and highly refractory to conventional therapies [1,2]. Therefore, more effective drugs are urged to improve current treatment regimens. Apart from cell growth, DNA repair, invasiveness, and angiogenesis, PDAC cells are hallmarked by mutations in genes involved in metabolism [1,3]. New therapeutic strategies targeting metabolism are emerging as promising approaches to overcome chemoresistance [4]. However, inter- and intra-tumor heterogeneity, often result in different metabolic phenotypes also as a consequence of multiple interactions with the tumor microenvironment [5]. This poses therapeutic limitations and highlights the importance of preliminary metabolic characterizations of the tumor lineages, preparatory to the administration of effective drugs. We recently demonstrated that two pancreatic cancer cell lines, characterized by a different metabolic profile, produce a dissimilar response to glucose deprivation/galactose substitution, an approach that is able to rewire energy metabolism [6]. Moreover, our group had already demonstrated the efficacy of dichloroacetate (DCA), an inhibitor of pyruvate dehydrogenase kinase (PDK), to kill cultured cells derived from human oral carcinomas, an effect inversely correlated with the mitochondrial respiratory capacity of the tumor cells [7]. Several in vivo and in vitro studies describe the ability of DCA to increase mitochondrial oxidative phosphorylation (OxPhos), reverting the Warburg effect and selectively targeting tumor cells [8,9]. As well, an extensive body of literature demonstrates the efficacy of DCA to enhance chemo-sensitivity in several cancer types [10,11]. DCA treatment has been purposed both for in vitro and in vivo studies also in pancreatic cancer [8,12,13,14]. Nevertheless, further investigations are necessary to better define the efficacy of the drug in this cancer type, to clarify potential additional mechanisms leading to cell death, and to explore possible further ways to limit the side effects encountered. In this study, we analyzed the effects of DCA on the two PDAC cell lines, PANC-1 and BXPC-3, chosen among others for their similar growth conditions and well-characterized geno-/phenotype [6,15,16]. A broad metabolite and transcriptome profiling of PDAC cell lines identified three tumor subtypes, with PANC-1 and BXPC-3 belonging to a lipogenic cluster hallmarked by a distinct reliance on glucose oxidation and mitochondria-related metabolism [17]. Testing DCA on 2D and 3D cultures of the PDAC cell lines, we demonstrated that the drug negatively affects vital parameters by reducing the mitochondrial respiratory activity and, most notably, the cancer stem cell compartment. Moreover, we showed that DCA is also able to mitigate in vivo tumor growth in a model of PDAC xenografted mice.

## 2. Materials and Methods

### 2.1. Cell Culture

PANC-1 and BXPC-3 cells were purchased from the American Type Culture Collection (ATCC, Manassas, VA, USA) and cultured at 37 °C in a 5% CO_2_ humidified atmosphere in complete RPMI medium supplemented with 10% fetal bovine serum, penicillin–streptomycin (100 U/mL), and 2 mM of glutamine, and the glucose concentration was typically 10 mM or 1 mM when indicated. Dichloroacetate (DCA) was purchased from Sigma-Aldrich (St. Louis, MO, USA). For each in vitro experiment, cells were treated with DCA 4 mM and 10 mM at indicated times.

### 2.2. Cell Growth Curves

Cell growth curves were performed as previously described [18].

### 2.3. Real-Time Cell Proliferation Monitoring by xCELLigence System

The xCELLigence experiments were performed using the RTCA (real-time cell analyzer) instrument, according to the manufacturers’ instructions (ACEA Biosciences, San Diego, CA, USA). The optimal seeding number was previously determined by cell titration and growth experiments (data not shown). The 2500 cells/well were then seeded and their proliferation was automatically monitored every 30 min and 24 h after seeding, cells were treated with DCA. The cell index was monitored up to 90 hours from seeding. Data were analyzed using xCELLigence software (Version 2.0, Acea biosciences, San Diego, CA, USA) and expressed as a mean ± SD of the cell index normalized to the last cell index recorded before the time of DCA addition.

### 2.4. Apoptosis Assay

After incubation with DCA, cells were stained with Annexin-V-FITC and PI (BD Biosciences). Live, apoptotic, and necrotic cells were detected using flow citometry (Navios, Beckman Coulter, Brea, CA, USA). Three independent experiments were carried out. A total of 10^4^ events for each sample were acquired.

### 2.5. Migration Assay

The effects of DCA on PANC-1 and BXPC-3 migration capacities were assessed using a scratch wound assay. Briefly, cells were seeded into six-well culture plates and cultured to complete confluence. Subsequently, three parallel, linear wounds were produced in each dish with a 200 μL plastic pipette tip. The cells were then treated with DCA and the wound healing ability, monitored at different time points, was quantified after 48 h. Three representative images of scratched areas from each dish were photographed to estimate the migration of cells. The cell migration rate was calculated using the following formula: [1 − (48 h scratch width/0 h scratch width)] × 100%. 

### 2.6. Lactate Measurements

A lactate colorimetric assay kit (Abcam, Cambridge, MA, USA) was used following the manufacturer’s protocol and the lactate concentration detected (intracellular or released) was normalized to cell number.

### 2.7. Metabolic Flux Analysis and Mitochondrial Respiratory Complex Enzymatic Activity

The oxygen consumption rate (OCR) and extra-cellular acidification rate (ECAR) were measured in adherent PANC-1 and BXPC-3 cells with an XF96 extracellular flux analyzer (Seahorse Bioscience, Billerica, MA, USA) as previously described [19]. Briefly, for the OCR analysis, after measuring basal respiration, oligomycin (1 μM), FCCP (1 μM), and rotenone + antimycin A (1 μM + 1 μM) were injected into each well sequentially to assess, respectively, the coupling of the respiratory chain, and the maximal and non-mitochondrial oxygen consumption. For the ECAR analysis, glycolytic flux (basal glycolysis, glycolytic capacity, and glycolytic reserve) was analyzed by the sequential addition of 10 mM glucose, 1 μM oligomycin, and 100 mM 2-deoxyglucose. The OCR and ECAR values were normalized to protein content in each well, determined using BCA assay (Thermo Scientific, Waltham, MA, USA).

### 2.8. Mitochondrial DNA Quantification

The measurement of mtDNA copy number, relative to nuclear DNA copy number, was determined as previously described [6].

### 2.9. Live Cell Imaging of mtΔΨ and ROS

Cells cultured at low density on fibronectin-coated 35-mm glass-bottom dishes (Eppendorf, Amburgo, Germany) were incubated for 20 minutes at 37 °C with 2 μM of TMRE, 10 μM of DCF (Molecular Probes, Eugene, OR, USA) to monitor mtΔΨ and ROS, respectively. Stained cells were washed with PBS and examined using a Leica TCS SP8 confocal laser scanning microscope. Acquisition, storage, and data analysis were performed with a dedicated instrumental software from Leica (LAS-X, Wetzlar, Germany).

### 2.10. Western Blotting Analysis

Aliquots, containing 40 μg of proteins from each lysate cell, were subjected to SDS polyacrylamide gel electrophoresis and transferred to a polyvinylidene difluoride membrane using a Trans Blot Turbo Transfer System. Membranes (Bio-Rad Laboratories, Hercules, CA, USA) were probed with the following primary antibodies: pyruvate dehydrogenase E1-alpha (PDH) and pPDH^Ser293^ (1:500, Abcam, Cambridge, UK), LC3B (1:1000 Cell Signaling Technology), TOM20 (1:1000, Santa Cruz Biotechnology, Santa Cruz, CA, USA), DRP1 (1:1000, BD Bioscences), OPA-1 (1:1000, BD Bioscences), MFN1 (1:1000, Santa Cruz), MFN2 (1:1000, Abnova, Tapei, Taiwan), and CASPASE 3 (1:1000, Cell Signaling Technology, Danvers, MA, USA). After incubation with a correspondingly suited horseradish peroxidase-conjugated secondary antibody (1:2500; Cell Signaling Technology), signals were developed using the enhanced chemiluminescence kit (ClarityTM Western ECL Substrate, Bio-Rad) and the ChemiDoc imaging system XRS + (BioRad), and then analyzed using Image Lab software (version 4.1, Bio-Rad, Hercules, CA, USA). The intensity of LC3B-II (corresponding to the cleaved fraction), TOM20, DRP1, OPA-1, MFN1, and MFN2 bands were normalized to the β-actin signal while PDH phosphorylation was normalized to total proteins.

### 2.11. Flow Cytometric Detection of Surface Markers

Surface marker expressions CD44, CD24, and EPCAM, was evaluated by citofluorimetric analysis in PANC-1 and BXPC-3 treated with DCA for 24 h. In brief, after trypsinization, cells were incubated in the dark at room temperature for 15 min with CD44-APC, EPCAM-FITC, and CD24-PE directly conjugated monoclonal antibodies (BDB). Cytofluorimetric analysis was performed by Navios (Beckman Coulter). The emitted fluorescent signal of 10,000 events for each sample was acquired and analyzed using the Kaluza Analysis software (version 1.3, Beckman Coulter, Brea, CA, USA).

### 2.12. Reverse Transcription and Real-Time PCR Analysis

One microgram of total RNA, isolated using Trizol reagent (Life Technologies, Paisley, UK), according to the manufacturer’s instruction and quantified on a Nanodrop spectrophotometer (Thermo Fisher Scientific, Waltham, MA, USA), was used in a reverse transcription (RT) reaction using the Transcriptor first strand cDNA synthesis kit (Roche Diagnostic, Penzberg, Germany) according to the manufacturer’s instructions. Quantitative real-time polymerase chain reaction (PCR) was performed in duplicate, using the QuantiTect Primer Assay (Qiagen, Basel, Switzerland) detecting Lin28 mRNA. Quantification of the mRNA levels was performed on a LightCycler^®^ 480 real-time PCR instrument. The relative amounts of Lin28 were normalized with GAPDH expression by Light Cycler^®^ 480 Software version 1.5 (ROCHE) using the 2ΔΔCt method.

### 2.13. 3D Culture

PANC-1 and BXPC-3 cells were detached with trypsin-EDTA and counted. Then, 1000 cells/well were seeded into ultra-low attachment 96-well round-bottomed plates and cultured in RPMI. To evaluate the DCA effect of preformed spheroids, 3D cultures were maintained for 7 days, obtaining spheroids. Then, medium was replaced with fresh medium and spheroids were treated with DCA 4 mM and 10 mM for 72 h. To evaluate the effect of DCA to spheroid formation, DCA was added to cell suspension when seeded into ultra-low attachment plates and the culture was maintained for 7 days. Spheroids were photographed on an inverted optical microscope (Axio Vert A1, Zeiss, Oberkochen, Germany) and their diameter was measured using the ZEISS ZEN imaging software. Spheroid viability was assessed using an MTS assay. A solution of cellTiter 96^®^ Aqueous MTS Reagent Powder (Promega, Madison, WI, USA) and PMS (Sigma Aldrich, Saint Louis, MO, USA) was added to each well of 3D spheroids culture. After 2 h of incubation at 37 °C the absorbance at 490 nm was measured and the percentage of viability in each well was calculated using the untreated spheroids as 100%. 

### 2.14. Animal Studies

The animal testing was executed in an AAALAC (Association for Assessment and Accreditation of Laboratory Animal Care International, Frederick, MD USA) accredited experimental facility under the approval number ANM14_002/468862. A total number of 5 × 10^6^ BxPC-3-luc cancer cells were cultured, resuspended in 0.1 mL of PBS/matrigel mixture (1:1) and then s.c. injected into the right flank of 5–6 weeks old Nu/Nu nude mice. When tumor size reached an average volume of 100 mm^3^, BxPC-3-luc tumor-bearing nude mice were randomly assigned into 2 groups (6 mice/group). Group 1 (normal saline, i.p, qw), group 2 (DCA, mg/kg, i.p, qw). Animals had free access to water. DCA was dissolved to generate a final concentration of 100 mg/kg/day (s.c: sub-cutaneous; i.p: intraperitoneal; qw: once a week). 

### 2.15. Statistical Analysis

Experimental data are expressed as the mean ± standard error mean (SEM) or mean ± standard deviation (SD). Data were compared using the unpaired Student’s *t*-test or one-way Anova, followed by the Bonferroni test. A *p* value < 0.05 was accepted as statistically significant.

## 3. Results

### 3.1. DCA Negatively Affects Cell Proliferation, Survival, and Migration in PANC-1 and BXPC-3 Cell Lines

The two PDAC cell lines selected for this study were PANC-1 and BXPC-3. PANC-1 is a pancreatic carcinoma-derived cell line of ductal cell origin. It can metastasize but has poor differentiation ability and harbors mutations in KRAS and TP53 and homozygous deletion in CDKN2A/p16 [16]. The BxPC-3 is a primary adenocarcinoma-derived cell line with moderate differentiation and epithelial morphology. It expresses mucin and high levels of angiogenic factors and cancer stem cell markers [16,20] and lacks KRAS mutations but harbors mutations in TP53 and homozygous deletions in CDKN2A/p16 and SMAD4/DPC4 [16].

**Figure 1:**
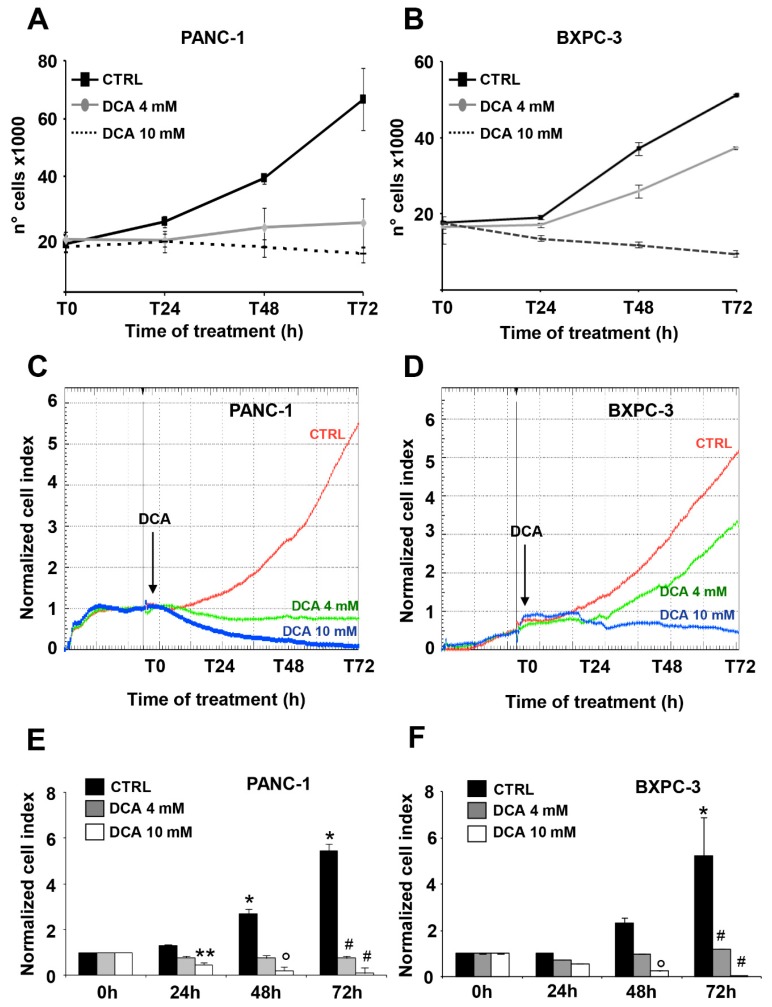
Effect of dichloroacetate (DCA) on cell growth and proliferation. Cell growth curves of cultured PANC-1 (**A**) and BXPC-3 (**B**) seeded at the same density, cultured for 72 h without CTRL (black line) or with 4 mM DCA (gray line) and 10 mM DCA (dotted line). Cells were counted every 24 h and the values shown are means ± SEM of three independent time courses. Dose- and time-dependent effect of DCA treatment on PANC-1 (**C**) and BXPC-3 (**D**) proliferation assessed using xCELLigence system. A representative profile for both cell lines is shown, indicating the cell index normalized to the last cell index recorded before DCA addition, measured in real time for 72 h. Normalized cell index quantified in PANC-1 (**E**) and BXPC-3 (**F**) cells treated with DCA indicated as mean ± SD of three independent experiments, recorded at the indicated time points. Analysis was conducted using one-way Anova and Bonferroni post-test. * *p* < 0.001 vs. CTRL; ** *p* < 0.05 vs. CTRL 24h; ° *p* < 0.001 vs. CTRL 48 h; # *p* < 0.001 vs. CTRL 72 h.

The effect of DCA on viability parameters in PANC-1 and BXPC-3 cell lines was assessed at the concentrations of 4 mM and 10 mM, already tested and proven effective as shown in our previous study [7]. First, we performed a cell growth assay for 72 h, which revealed a significant dose- and time-dependent sensitivity of both cell lines to the DCA treatment (Figure 1A,B). In particular, PANC-1 and BXPC-3 displayed a similar block of cell growth when treated with 10 mM DCA starting from the first day of incubation, and conversely, at the lower dose of 4 mM tested the PANC-1 cell line appeared significantly more sensitive to the drug. 

The above reported observation, particularly interesting because of the well-known chemoresistance shown by the PANC-1 cell line [21,22], prompted us to verify the DCA-mediated cell growth inhibition by a different approach. To this aim, we monitored in real time the dynamic changes in cell proliferation and viability by impedance-based technology. As shown in Figure 1C–F, 10 mM DCA treatment drastically depressed cell proliferation in both cell lines whereas 4 mM DCA treatment caused a much stronger inhibitory effect in PANC-1 as compared with the BXPC-3 cells lines. To note, the effects of DCA were clearly visible as early as after 24 h of incubation with the drug. Real-time cell growth analysis was also carried out with low glucose in the culturing medium (i.e., 1 mM in RPMI). As expected, the growth rate of both of the PDAC cell lines was severely dampened given their metabolic dependence on glucose oxidation [17]. However, the different sensibility with the 4 mM DCA treatment was confirmed also with a low glucose regimen (Supplementary Figure S1). 

**Figure 2:**
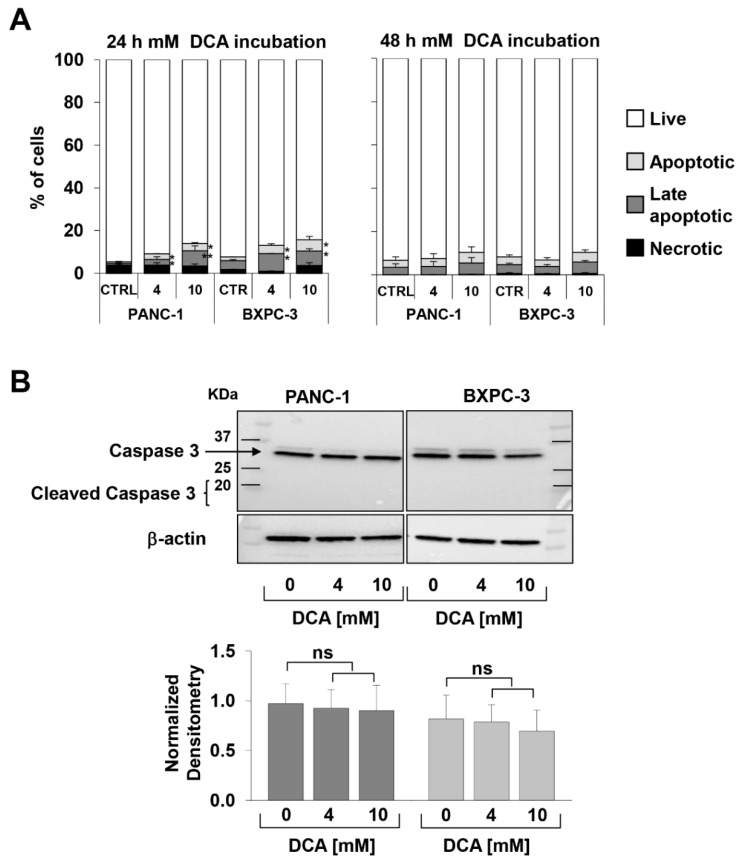
Effect of DCA on vital parameters and cell migration capacity, (**A**) measurement of early and late apoptotic and necrotic cells was performed by flow-cytometry as described in Material and Methods, cells were treated with 4 and 10 mM DCA for 24 h (left panel) and 48 h (right panel), data are expressed as percentage of total events analyzed and are the mean ± SEM of at least three independent experiments (* *p* < 0.05 vs. CTRL, ** *p* < 0.01 vs. CTRL); (**B**) expression of caspase 3, (upper panel) representative western blotting of total protein lysate, (lower panel) densitometric analysis (normalized to β-actin) of the uncleaved caspase 3 band, the bars are means ± SEM of three independent experiments under each condition; NS, not significant.

To evaluate vital parameters, we used the annexin V-FITC/PI assay and assessed by flow cytometry the relative amount of necrotic, late, and early apoptotic cells. The results obtained showed that after 24 h incubation with DCA both PANC-1 and BXPC-3 cell lines displayed a slight but significant dose-dependent increase of apoptosis as compared with untreated cells. However, the amounts of apoptotic cells were relatively low (i.e., < 10% at the higher concentration of DCA tested) and did not increase further at 48 h of DCA treatment (Figure 2A). Accordingly, the expression of uncleaved caspase 3 did not change following DCA treatment and no appreciable amount of its cleaved form was detectable (Figure 2B). This result suggested a cytostatic rather than a cytotoxic activity of the drug in both cell lines to account for the marked dampening of the growth rate shown in Figure 1A–D. 

Next, we evaluated the effect of DCA on the cell motility, performing the scratch wound-healing assay. The migration capacity, observed at different time points, was measured after 48 h upon 4 mM and 10 mM DCA treatment. Both PANC-1 and BXPC-3 cells decreased their motility when treated with the higher dose of DCA, whereas, BXPC-3 migration ability was not affected by 4 mM DCA treatment, which, instead, caused a delay in the wound closure capacity in PANC-1 cells, confirming their greater sensitivity to the drug detectable at a lower concentration (Supplementary Figure S2). 

### 3.2. DCA Alters the Energetic Cell Metabolism in PDAC Cell Lines

**Figure 3:**
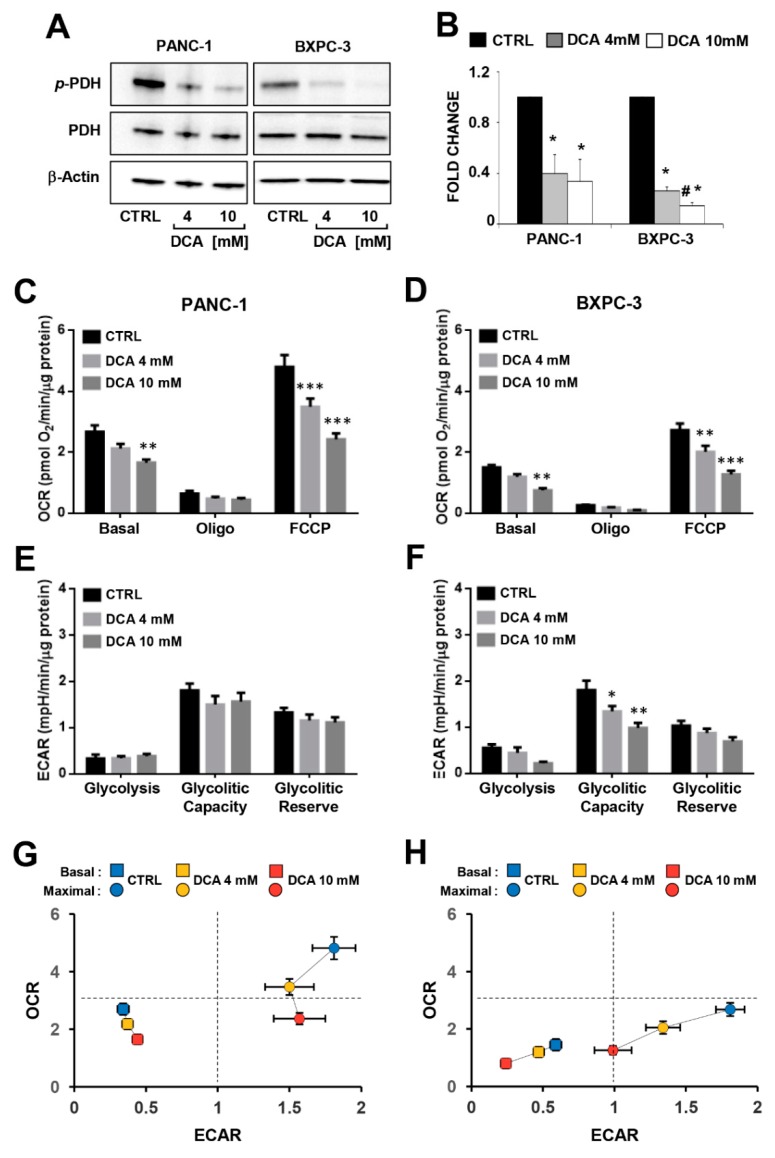
Effect of DCA on pyruvate dehydrogenase E1-alpha (PDH) phosphorylation and metabolic fluxes, (**A**) a representative immunoblotting of the phospho-S293 E1αPDH (pPDH) and total enzyme subunit in PANC-1 and BXPC-3 cells following 24 h incubation with DCA 4 mM and 10 mM, β-actin was used as loading control; (**B**) quantitative analysis of the densitometric values of phospho-S293 E1αPDH relative to total PDH, normalized to β-actin, the results are the means ± SEM of three independent experiments, * *p* < 0.05 vs. CTRL, # *p* < 0.05 vs. BXPC-3 DCA 4 mM; (**C**,**D**) oxygen consumption rate (OCR) performed by the SeaHorse platform as described in the Material and Method section and normalized to the protein content of the cells at the end of the assay, basal: resting OCR; oligo: OCR measured after the addition of the ATP synthase inhibitor oligomycin; FCCP: OCR measured after the uncoupler FCCP producing the maximal respiratory capacity, the OCR was corrected for the residual OCR measured after the addition of the CxI inhibitor rotenone (not shown); (**E**,**F**) extracellular acidification rate (ECAR) measured in PANC-1 and BXPC-3 treated with DCA 4 mM and 10 mM for 48 h, glycolisis: resting ECAR; glycolitic capacity: ECAR measure after the addition of olygomycin and FCCP and refers to maximal glycolytic activity with the OxPhos inhibited; glycolytic reserve: difference between ECAR measured in the presence of oligomycin + FCCP and under resting conditions, the bars represent the means ± SEM of 3 independent experiments carried out in 3 technical replicates under each condition (* *p* < 0.05, ** *p* < 0.01, *** *p* < 0.001 vs. respective CTRL); (**G**,**H**) energy maps obtained plotting basal and maximal ECAR and OCR capacities obtained in PANC-1 and in BXPC-3, respectively.

In order to investigate the link between the DCA-induced antiproliferative effect and alterations in the metabolism of the PDAC cell lines, we evaluated the efficacy of the compound to inhibit its recognized target pyruvate dehydrogenase kinase (PDK), assessing the phosphorylation state of the E1α subunit (residue S293) of the pyruvate dehydrogenase complex (PDC) by Western blotting in PANC-1 and BXPC-3. As shown in Figure 3A,B, the normalized level of P-PDH-E1 was significantly decreased in both cell lines while the expression level of total PDH was comparable, and, as expected, not modified by the drug treatment. Unexpectedly, both the extra- and intracellular lactate production was apparently unaffected in both the DCA-treated PDAC cell lines (Supplementary Figure S3). 

Then, we analyzed the major metabolic fluxes by measuring extracellular acidification and oxygen consumption using the SeaHorse technology. When the metabolic fluxes were assessed following 24 h incubation of DCA no significant changes were observed in both of the PDAC cell lines (Supplementary Figure S4). Longer (i.e., 48 h) exposure to DCA caused a dose-dependent decrease of the mitochondrial oxygen consumption rates (OCRs) in both of the PANC-1 and BXPC-3 cell lines under basal as well as in the presence of the ATP-synthase inhibitor oligomycin or the uncoupler FCCP (i.e., the maximal respiratory capacity) (Figure 3C,D). It should be noted that the mitochondrial respiratory activity was significantly higher in PANC-1 than in BXPC-3, indicating a more OxPhos-reliant metabolic phenotype. The extracellular acidification rates (ECARs), which are linked to the glycolytic flux, did not result in significant changes following the DCA treatment of PANC-1, while inhibition was observed for the basal ECAR in BXPC-3 at the higher concentration of DCA and dose dependently for the glycolytic capacity (Figure 3E,F). Consequently, the overall bioenergetic profiles of the basal and stimulated fluxes capacities of both PDAC cells was affected by DCA, with PANC-1 displaying a decrease in the OxPhos capacity and BXPC-3 showing a severer impairment of both the metabolic fluxes (Figure 3G,H). Measurement of the metabolic fluxes under low glucose growth condition resulted in reduced OCR in both cell lines (more consistent in BXPC-3) and in enhanced ECAR in BXPC-3. The DCA treatment (48 h) caused in both cell lines a significant lower inhibitory effect on OCR (particularly at 4 mM) at low glucose as compared with the high glucose regimen. No significant change was caused by the DCA treatment on ECAR in PANC-1 whereas a 40–50% inhibition was observed in BxPC-3, however, it was independent on the availability of glucose (Figure S5). 

All together, these unexpected observations indicated that in PDAC cells, though and notwithstanding DCA was apparently able to activate PDH, no reverse of the Warburg effect was attained. Instead, DCA treatment caused a bioenergetic crisis, leading to dampening of the cell growth by an overwhelming off-target mechanism. 

### 3.3. DCA Induces ROS Production in PDAC Cell Lines and Differentially Affects Mitochondrial Biogenesis and Dynamics 

**Figure 4:**
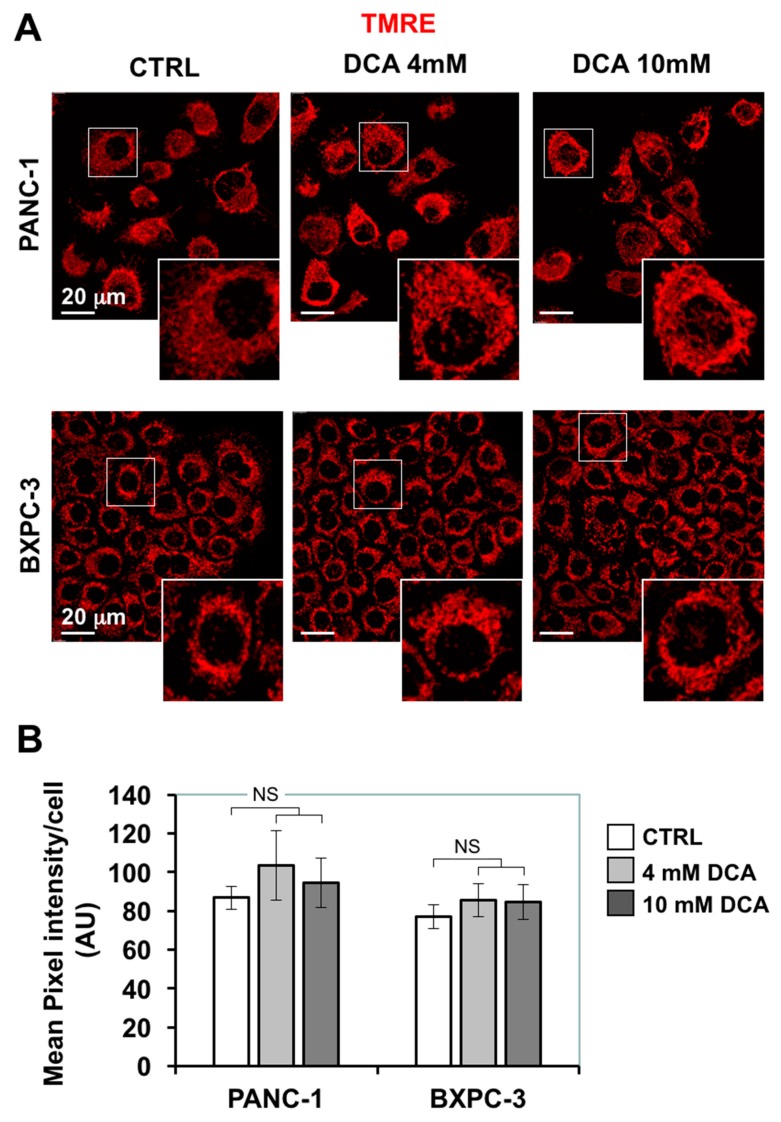
Effect of DCA on mitochondrial morpho-functional parameters in PANC-1 and BXPC-3 cell lines, (**A**) representative laser scanning confocal microscopy imaging of Δψ_m_ by the fluorescent probe TMRE in PANC-1 (upper panels) and BXPC-3 (lower panels), digital magnification of the TMRE-related fluorescence of the mitochondrial compartment is also shown for the details indicated by the squares (ImageJ software from http://imagej.nih.gov/ij/); (**B**) histogram showing the quantitative analysis of the TMRE-related fluorescence/cell, the values are means ± SEM of three independent experiments under each condition where the digitalized fluorescence images from at least five randomly selected optical fields (each containing about 25 cells) were analyzed; NS, not significant.

The depressed mitochondrial respiration caused by DCA led us to investigate further mitochondrial functions. First, we assessed the morpho-functional architecture of the mitochondrial compartment by confocal microscopy imaging using the fluorescent ΔΨ-probe TMRE which accumulates in respiring mitochondria. Figure 4A shows that in PANC-1 the TMRE-related signal displayed a diffused particulate appearance largely spread in the cytoplasm, indicative of a prevalent fragmented rather than interconnected structure. A similar feature resulted also in the smaller BXPC-3 cells, which, however, displayed an annular peri-nuclear compartmentalization. The 24 h treatment of DCA did not cause major changes either in the intensity of the TMRE fluorescent signal nor in its morphological appearance. 

**Figure 5:**
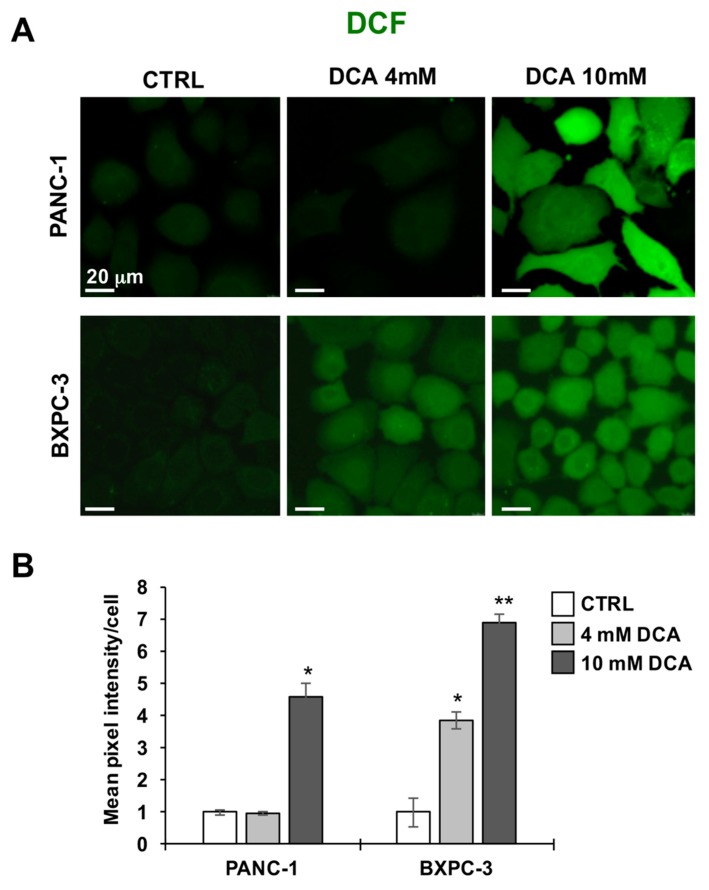
Effect of DCA on ROS production in PANC-1 and BXPC-3 cell lines, (**A**) representative LSCM imaging of ROS production in living PANC-1 (upper panel) and BXPC-3 (lower panel) cells treated with DCA assessed by DCF, images are representative of three different preparations yielding similar results; (**B**) histogram showing the quantitative analysis of the DCF-related fluorescence/cell, the values are means ± SEM of three independent experiments under each condition where the digitalized fluorescence images from at least five randomly selected optical fields (each containing about 25 cells) were analyzed; * *p* < 0.01, ** *p* < 0.001 vs. CTRL.

Next, we imaged the two PDAC cell lines for their redox tone using the peroxide probe DCF. Figure 5A,B show that the 10 mM DCA treatment for 24 h caused a significantly large increase of the DCF-related signal in both PANC-1 and BXPC-3 cell lines as compared with their untreated basal levels. This result indicated a pro-oxidative unbalance or the redox state caused by DCA exposure. 

**Figure 6:**
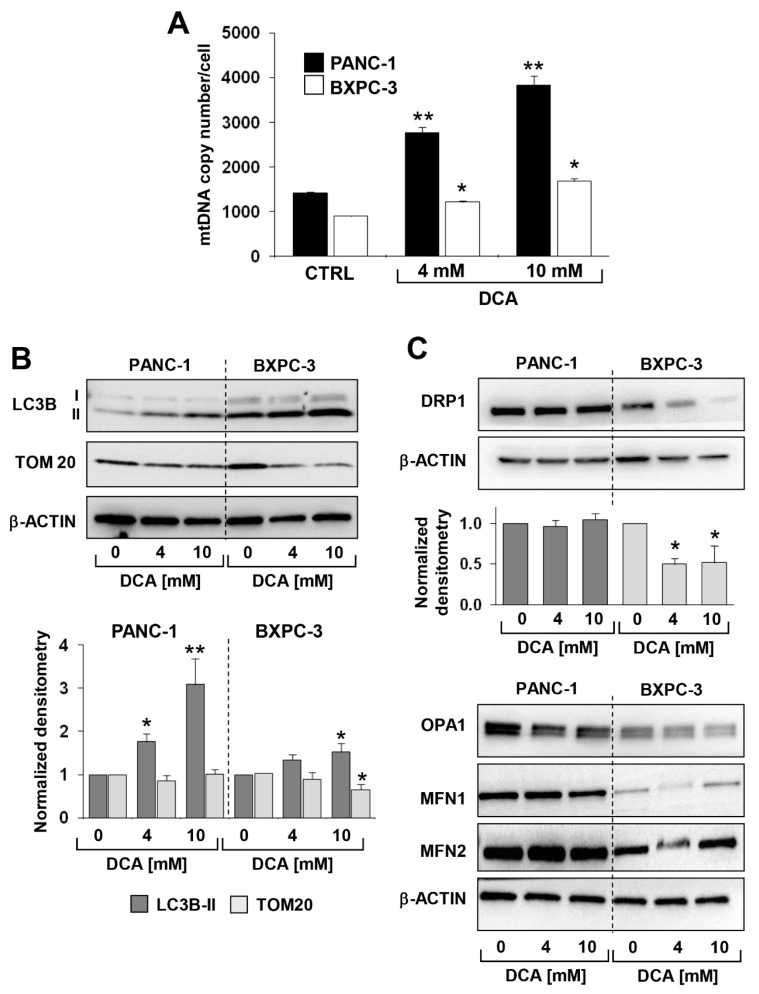
Effect of DCA on mitochondrial biogenesis, mitochondrial dynamics and mitophagy in PANC-1 and BXPC-3 cell lines, (**A**) mtDNA copy number assessed by q-RT-PCR, the bar histogram shows values normalized to the nuclear DNA and are the means ± SEM of three independent experiments (* *p* < 0.05 vs. PANC-1 CTRL, ** *p* < 0.05 vs. BXPC-3 CTRL); (**B**) representative immunoblotting for protein expression levels of LC3B and TOM20 in PANC-1 and BXPC-3 treated with DCA and densitometric analysis (lower histogram) performed for three independent experiments and expressed as mean ± SEM (* *p* < 0.05, ** *p* < 0.05 vs. respective CTRL 0 mM DCA); (**C**) representative immunoblotting for protein expression of factors involved in mitochondrial fission (top panel) and fusion (bottom panel), upper panel shows a representative immunoblotting for protein expression level of DRP1 with densitometric analysis (* *p* < 0.05 vs. CTRL-0 mM DCA), lower panel shows a representative immunoblotting for OPA-1, MFN1, MFN2, and the β−actin.

The above reported analysis was complemented with the measurement of the mitochondrial DNA (mtDNA). As shown in Figure 6A, the mtDNA copy number/cell was significantly higher in PANC-1 than in BXPC-3, consistently with the more active respiratory activity. Following the DCA treatment, a progressive dose-dependent increase of the mtDNA was observed in both of the PDAC cell lines. This was likely due to a compensatory response to the OxPhos dysfunction caused by DCA. 

Next, we assessed by immunoblotting the expression level of proteins known to be involved in the mitochondrial clearance (i.e., mitophagy) and dynamics. Figure 6B,C show that the expression of the autophagosome marker LC3B-II was significantly higher in BXPC-3 than in PANC-1, and that DCA caused a dose-dependent progressive increase of the marker in both cell lines. However, TOM20, a marker of the outer mitochondrial membrane, significantly decreased only in the BXPC-3 cell line. 

Analysis of the factors involved in the fusion/fission processing of mitochondria revealed that all were expressed at higher levels in PANC-1 as compared with BXPC-3 but with a differential effect on them caused by the DCA treatment (Figure 6C). In particular, only DRP1, a factor involved in mitochondrial fission, decreased following the DCA treatment in BXPC-3. No significant change in the DRP1 expression was detected in PANC-1 as well as in the mitochondrial fusion-inducing factors OPA1, MFN1, and MFN2 in both cell lines following the DCA treatment (Supplementary Figure S6).

Taken together, these observations suggest a more dynamic profile of the mitochondrial compartment in PANC-1, which undergoes active mitochondrial fusion-fission, with an apparently low level of mitophagy processing. Conversely, BXPC-3 cells appear more phenotypically prone to pursue the organelle quality control. This might be consistent with the more fragmented appearance of the mitochondrial network in BXPC-3 cells. The DCA treatment caused an evident enhancement of the mitophagic marker LC3B in both cell lines, which, however, remained much larger in BXPC-3. 

### 3.4. DCA Differentially Affects the Cancer Stem Cell Compartment in PDAC Cell Lines 

**Figure 7:**
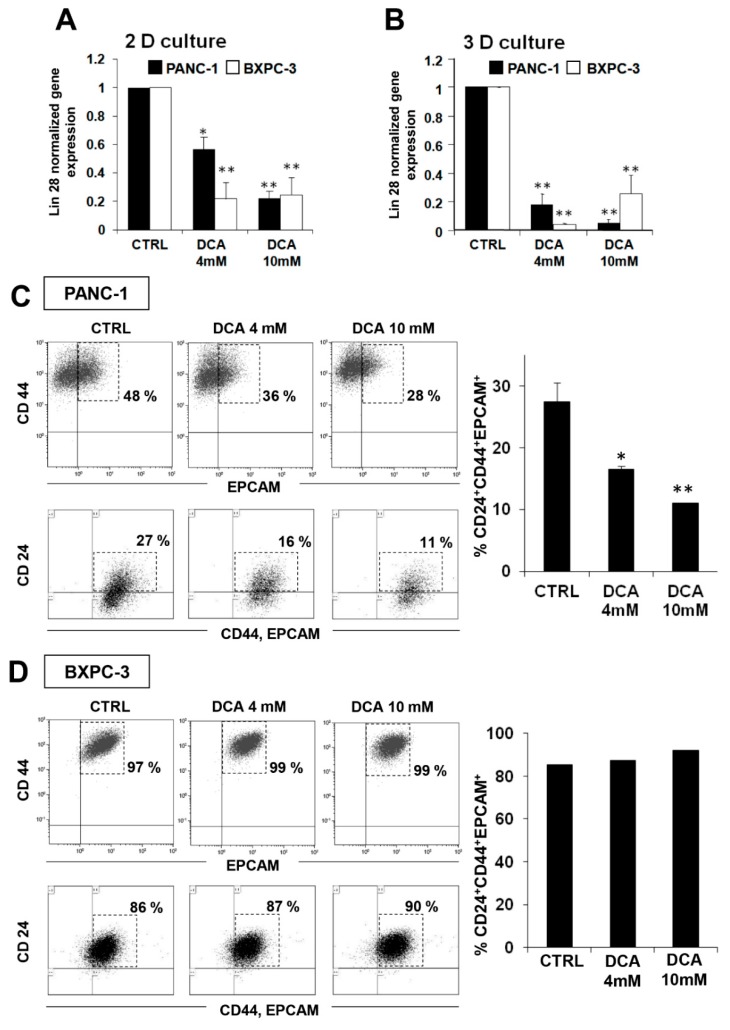
Effect of DCA on PANC-1 and BXPC-3 cancer stem cell markers, (**A**) quantitative RT-PCR analysis of Lin28 transcript performed in PANC-1 and BXPC-3 grown in monolayer for 24 h with DCA 4 mM and 10 mM, the values are the mean ± SEM of three independent experiments (* *p* < 0.05 vs. CTRL, ** *p* < 0.01 vs. CTRL); (**B**) quantitative RT-PCR analysis of Lin28 transcript performed in PANC-1 and BXPC-3 spheroids cultured for 7 days and treated with DCA for 72 h, the values are the mean ± SEM of three independent experiments (** *p* < 0.01 vs. CTRL); (**C**) representative dot plot analysis of CD44 (y axis) and EPCAM (x axis) expression in PANC-1 (upper tripartite panel) treated with DCA 4 mM and 10 mM for 24 h, the lower tripartite panel shows the corresponding expression of CD24 in CD44^+^/EPCAM^+^ cells; (**D**) representative dot plot analysis of CD44 (y axis) and EPCAM (x axis) expression in BXPC-3 (upper tripartite panel) treated with DCA 4 mM and 10 mM for 24 h, the lower tripartite panel shows the corresponding expression of CD24 in CD44^+^/EPCAM^+^ cells; the percentage in each dot plot of (**C**) and (**D**) of CD44^+^/EPCAM^+^ and CD24^+^/CD44^+^/EPCAM^+^ cells is shown; the histograms on the right of (**C**) and (**D**) show the mean ± SEM of CD24^+^/CD44^+^/EPCAM^+^ positive cells (expressed as percentage of total analyzed events) of at least three experiments; * *p* < 0.05, ** *p* < 0.01 vs. CTRL.

To investigate possible additional mechanisms of the cytostatic effect of DCA, we decided to test its impact on the cancer stem cell (CSC) fraction of the PDAC cell lines. To this aim, we evaluated the expression of the embryonic stem cell factor Lin28 proven to be involved in cell metabolism [23] and known to be a biomarker for poor prognosis of cancer progression [24,25]. Figure 7A shows that 48 h of the DCA treatment caused a significant reduction of the Lin28 expression in both cell lines with BXPC-3 appearing more sensitive than PANC-1. A similar result was obtained in 3D cultures obtained from both cell lines (Figure 7B). 

This observation prompted us to deepen the effect of DCA on CSCs by FACS analysis of the specific pancreatic CSC surface markers CD24, CD44, and EPCAM [26,27]. Figure 7C shows a significant dose-dependent reduction of CD24^+^/CD44^+^/EPCAM^+^ cells in PANC-1 cells treated for 48 h with DCA. Of note, the fluorescence intensity was almost 50% reduced upon 4 mM DCA treatment in PANC-1 cells while at 10 mM, a stronger reduction of fluorescent intensity was already observed after 24 h treatment and did not change after 48 h treatment (data not shown). On the contrary, although CSC markers were expressed in about 90% of the BXPC-3 cells, their expression seemed to be not affected by the DCA treatment (Figure 7D). It has been recently reported that, more than the absolute expression level of given markers, it is their ratio to identify the subpopulation with more genuine stemness features. In particular the CD44/CD24 ratio appears to be the more reliable marker of CSC in tumorigenesis and metastasis [28]. In keeping this notion, it is relevant that the CD44/CD24 expression ratio in PANC-1 resulted 8-fold higher that in BXPC-3 (Supplemental Figure S7), thereby indicating that, although less populated, the CSC compartment is qualitatively more stem cell like. Conversely, the low expression level of the CSC markers broadly spread in the BXPC-3 population would phenotype them as early progenitors. 

### 3.5. Effect of DCA on 3D Cultures

**Figure 8:**
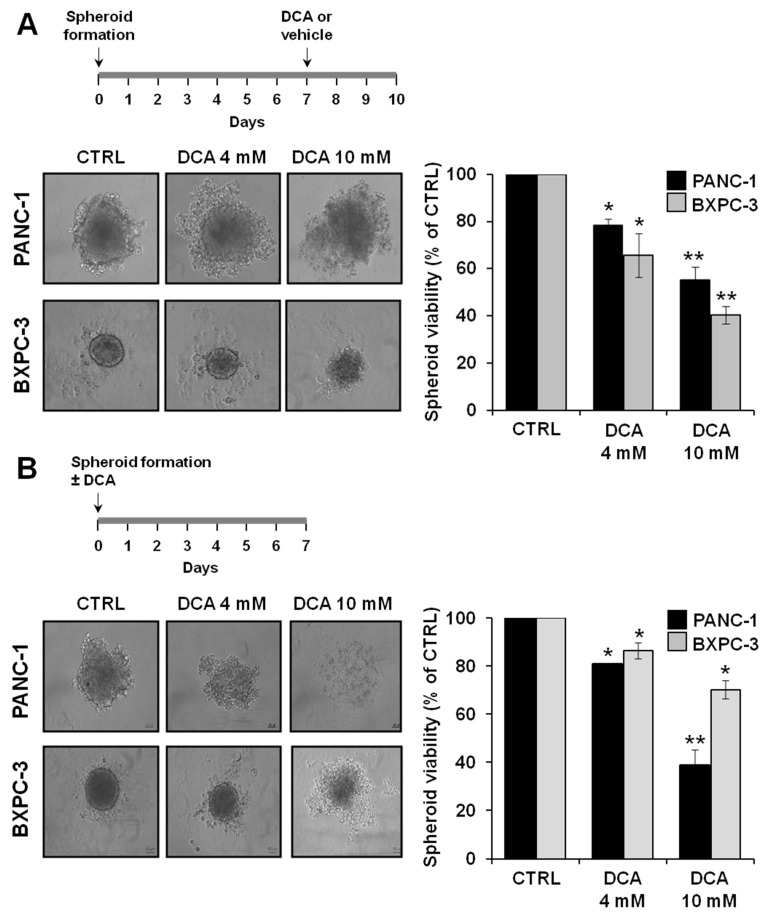
Effect of DCA on 3D culture from PANC-1 and BXPC-3 cells, (**A**) representative images of 3D culture obtained from PANC-1 (upper panel) and BXPC-3 (lower panel) seeded into ultra-low attachment plates, cultured for 7 days to obtain spheroid and treated with DCA 4 mM and DCA 10 mM for 72 h, spheroid viability (expressed as a percentage value compared to untreated condition) measured by MTS assay, * *p* < 0.05, ** *p* < 0.01 vs. CTRL; (**B**) inhibition of spheroid formation from PANC-1 and BXPC-3 cells treated with 4 mM DCA and 10 mM DCA treatment for 7 days, cell suspension of 1000 cells was seeded into ultra-low attachment plates and treated with DCA, spheroid viability (expressed as a percentage value compared to untreated condition) was measured by MTS assay; * *p* < 0.05, ** *p* < 0.01 vs. CTRL.

To further investigate the antitumor properties of DCA, we analyzed its biological effects on an alternative test bed constituted by 3D cultures of PANC-1 and BXPC-3. A mounting body of evidences suggests that cancer cell-derived spheroids are enriched in CSCs or cells with stem cell-related characteristics [29,30]. Figure 8A shows micrographs of the spheroids attained after 7 days of culture from which it can be clearly noted a difference in size and compactness of the boundary layer between PANC-1 and BXPC-3 cell lines. In particular, the spheroids derived from the PANC-1 cells appeared larger and with irregular borders as compared with those derived from the BXPC-3 cells. Treatment with DCA for 72 h altered the spheroids morphology which became progressively less defined. This was particularly evident in the PANC-1-derived spheroids at 10 mM DCA treatment. Consistent with this observation, a progressive dose-dependent reduction of cell viability in the spheroids of PANC-1 and BXPC-3 was clearly detectable. 

We also investigated the ability of DCA to affect spheroid formation by treating cell suspension at seeding. As shown in Figure 8B, DCA strongly affected spheroid formation in both of the PDAC cell lines, with PANC-1 resulting more sensitive to the drug. Accordingly, cell viability of PANC-1 spheroids was significantly more affected than that of BXPC-3 spheroids. 

### 3.6. DCA Mitigates Cancer Progression in A PC Xenograft Mouse Model

**Figure 9:**
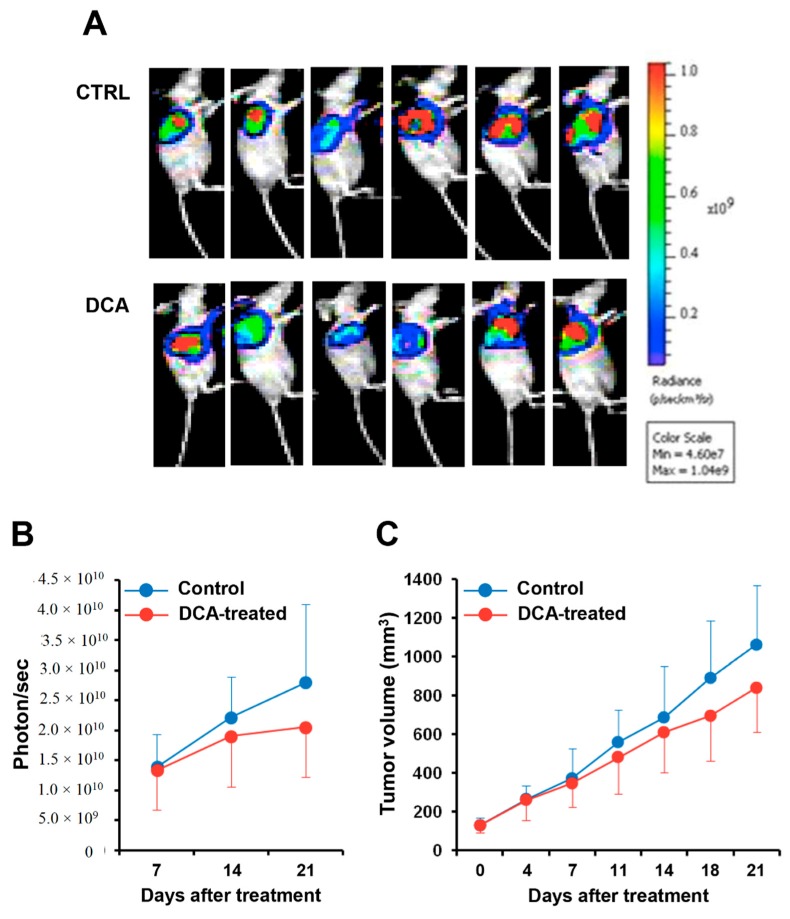
In vivo effect of DCA on PDAC tumor. Bioluminescence signaling was measured as photons/sec in CTRL group 1 receiving normal saline, i.p., qw, and in DCA group 2 receiving DCA (**A**,**B**). The tumor masses were harvested and the tumor volume was measured (**C**). See Materials and Methods for details.

We finally evaluated the effect of DCA administration in a xenograft pancreatic cancer mouse model. Luciferase-expressing BXPC-3 cell line was injected into nude mice and after reaching a volume of 100 mm^3^ were treated with DCA or vehicle for three weeks. Figure 9A shows the bioluminescence imaging of the tumor mass in DCA-treated and control mice. Quantification of the bioluminescence signal revealed a retarded progression of the pancreatic tumor in the DCA-treated mice documented by a 25–30% reduction both of the intensity of the bioluminescence signal of the tumor mass and of its volume as compared with vehicle-treated mice (Figure 9B,C). However, because of the large interindividual variability the differences did not reach statistical significance. 

## 4. Discussion

Aberrant metabolic processes generally occur in cancer cells, and therefore targeting metabolism represents an emerging strategy to treat tumors, including pancreatic cancer [31,32,33]. Tumor heterogeneity may result in malignant cells with distinct metabolic phenotype, and consequently, different sensitivity to metabolic drugs as in the case of gemcitabine toward the majority of patients with pancreatic cancer develop resistance [17,18,19,20,21,22,23,24,25,26,27,28,29,30,31,32,33,34].

In the present study, we tested the efficacy of the metabolic drug DCA in 2D and 3D cultures from two different well-characterized pancreatic cancer cell lines (i.e., PANC-1 and BXPC-3) as well as in a xenograft model of pancreatic cancer. Both cell lines, grown in monolayer, showed a marked sensitivity to DCA at the highest concentration tested (i.e., 10 mM), which halted cell proliferation and severely inhibited their migration capacity. Testing the drug at a lower concentration (i.e., 4 mM DCA) highlighted a higher sensitivity of the PANC-1 cell line. This observation is of interest because PANC-1 is reported as an aggressive and chemo-resistant cell line [21,22,35]. 

Analysis of the viability parameters in both the DCA-treated PDAC cell lines unveiled a limited percentage of apoptotic/necrotic cells, thereby suggesting a cytostatic rather than a cytotoxic effect exerted by the drug, confirming previous reports [36,37].

A major effect of DCA is generally attributed to its ability to induce a metabolic switch from glycolysis to mitochondrial glucose oxidation. This is achieved by inhibition of the PDH kinase PDK, thereby shifting PDH toward its more active unphosphorylated state [38,39]. Consequently, pyruvate is converted into acetyl-CoA which enters the tricarboxylic acid cycle and fuels the mitochondrial oxidative phosphorylation. 

However, in this study, we found that despite a substantial DCA-induced dephosphorylation of PDH no activation of the mitochondrial respiratory activity was observed in both of the drug-treated PDAC cell lines. On the contrary, DCA caused a dose-dependent reduction of mitochondrial OxPhos which was coupled to inhibition of the glycolytic capacity in BXPC-3. 

This result was also somewhat surprising considering that in a previous study with oral cancer cell lines we demonstrated that PE15 cells, characterized by a sustained OxPhos, were resistant to the DCA treatment, while HSC2/3 cells, exhibiting a glycolytic profile, appeared more drug-sensitive with a marked effect also on mitochondrial morpho-functional parameters [7]. Moreover, in another study with PANC-1 and BXPC-3 cell lines, we demonstrated a differential sensitivity to glucose deprivation/galactose substitution, a condition that also fosters the oxidative metabolism, with the more glycolytic BXPC-3 cells being more vulnerable [6]. This led us to hypothesize that the dissimilar sensitivity of different cell lines to drugs or conditions promoting a pro-oxidative metabolic shift was dependent on their basal metabolic profile, with those relying more on glycolysis and/or with low respiratory capacity being more vulnerable. 

The depressing effect of DCA on mitochondrial respiration did not apparently result in changes of the mitochondrial morphology, although a significant reduction of the fission promoting-factor Drp1 was observed in DCA-treated BXPC-3 cells. Likely the basal fragmented phenotype of the mitochondrial network in BXPC-3 hidden to appreciate further mitochondrial fragmentation. However, the mtDNA copy number/cell was significantly increased in both the PDAC cell lines, likely due to a compensatory mechanism as a consequence of mitochondrial dysfunction leading to activation of mitophagy, as demonstrated by the increased cleaved form of LC3B-II. The observed enhanced production of ROS in DCA-treated PDAC cell lines might elicit the organelle quality control to remove damaged mitochondria. Unbalance of ROS homeostasis is commonly related to dysfunction of the mitochondrial respiratory chain, although the relationship is often not clear (i.e., cause, effect, vicious cycle). Generally, considered a pro-survival mechanism protecting cells under stress conditions (oncogenic function) [40], more recently it has been demonstrated that dysregulation of mitophagy contributes to drug resistance (tumor suppressive role) [41]. Anyway, induction and inhibition of mitophagy in cancer progression is still controversial. 

All together the aforementioned observations do not enable us to rationalize the cytostatic effect of DCA as simply linked to metabolic rewiring of the PDAC cells. It must be taken into account that DCA can target other cellular pathways in addition to PDK. Indeed, it has been reported that DCA affects the CoA biosynthetic pathway [42], activates the AMPK signaling pathway [43], antagonizes with acetate [44], and disturbs the tyrosine catabolism [45]. Moreover, comparison of the metabolite profiles in cells treated with DCA or more selective novel inhibitors of PDK resulted in different outcomes [46]. This led us to investigate additional potential off-target effects of DCA to explain its efficacy to hit tumor cells. 

Cancer stem cells (CSCs) represent a fraction of the whole tumor mass emerging as responsible for cancer therapy refractory as well as metastasis dissemination and tumor relapse [47], thereby attracting growing interest as targets for the development of new anticancer therapies [48]. To the best of our knowledge, there is no report about the effect of DCA on pancreatic cancer stem cells. To dissect this intriguing aspect, first, we evaluated the effect of DCA treatment on Lin 28 expression revealing a significant dose-dependent downregulation detectable in both cell lines. Of note, Lin28 expression is strictly linked to metabolism since it is able to regulate cancer cell progression via PDK1 and to induce an energetic switch [49]. Lin28 is involved in the formation of CSCs [50] and its aberrant expression is associated with many human neoplastic diseases, including pancreatic cancer [51,52]. FACS analysis of the surface antigens expression CD44, CD24, and EPCAM typically featuring pancreatic CSCs [53] unveiled that DCA treatment reduced the percentage of the triple positive fraction in PANC-1. In contrast, we did not appreciate any DCA-induced modulation in BXPC-3 which were mainly constituted by triple positive cells. It should be taken into account that although more than 90% of the BXPC-3 was positive for stemness markers, their expression level was relatively low. Conversely, PANC-1 expressed higher levels of stemness markers, even though only in less than 30% of the cell population, suggesting a younger CSCs phenotype characterizing this cell subset. Consistent with this observation is the notion that rather than the absolute expression of the CSC markers it is their ratio to “qualify” the stemness propensity of cancer cells [28]. In keeping that the CD44/CD24 expression ratio in PANC-1 is much higher than in BXPC-3 this would indicate that, although less populated, the CSC compartment in PANC-1 is qualitatively more stem cell like. Conversely, the low expression level of the CSC markers broadly spread in BXPC-3 cell population would phenotype them as early progenitors. This difference in the two pancreatic cancer cell lines might account for their distinct metabolic phenotypes and sensitivity to chemotherapeutic drugs as well as to DCA. 

Three-dimensional (3D) cell culture technology has become a focus of research in tumor cell biology. Compared to 2D, 3D cultures from cell lines involve an enrichment in CSCs [30,54] and by mimicking the metabolic and proliferative gradients of in vivo tumors, provide a more reliable prediction of the response to a possible treatment [55,56]. In this perspective, we tested the efficacy of DCA on 3D culture obtained from PANC-1 and BXPC-3 and showed that DCA treatment compromised the structure and viability of already formed spheroids and compromised spheroid formation from both cell lines. In particular, at the higher dose DCA was able to almost completely inhibit spheroid formation from PANC-1. Consistent with our observations in 2D cultures, BXPC-3 spheroids were less sensitive then PANC-1 spheroids to DCA treatment. Likewise, in 2D cultures a significant Lin28 downregulation was also observed in spheroids from both cell lines. Although the above reported changes in the expression level of broadly recognized CSC markers in both 2D and 3D cultures do not imply conclusive evidence of the effect of DCA on the stemness compartment of PDAC, nevertheless, they provide hitherto unappreciated clues deserving further investigations. At the in vivo level, DCA treatment caused a slower, although not significant, cancer growth in BxPC-3-luc tumor-bearing mice as compared to the control mice, as assessed by reduced photon counting and decreased tumor volume.

**Figure 10:**
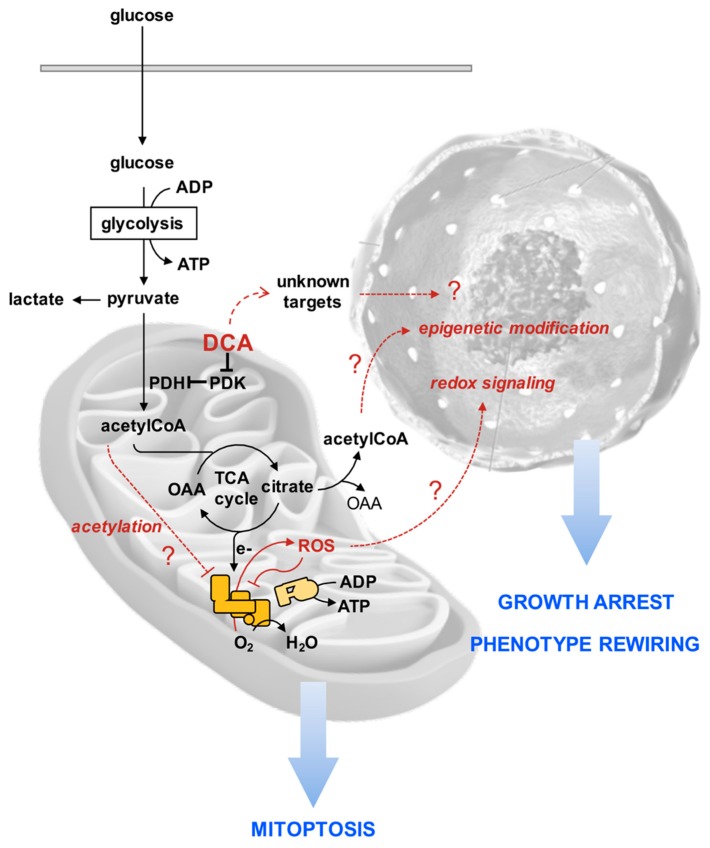
Working model for the effects of DCA described in this study. The pathway of the DCA-mediated mitochondrial oxidation of pyruvate is shown by black arrows. PDK: pyruvate dehydrogenase kinase; PDH: pyruvate dehydrogenase; TCA: tricarboxylic acid; OAA: oxaloacetate. The red lines and the question marks in the mitochondria and nucleus indicate hypothesized effects directly or indirectly linked to the DCA action. See Discussion for further details.

To rationalize the puzzling effects of DCA on the PDAC cell lines reported in our study, we put forward the following hypothetical sequence of events purposed to stimulate further investigations (Figure 10). We suggest that consequently to an enhanced oxidation of pyruvate more reducing equivalents are transferred to the mitochondrial respiratory chain with generation of ROS. The respiratory complexes are both producers and target of ROS [57], thereby fostering a vicious cycle leading to progressive inhibition of the functional electron transfer throughout the respiratory chain promoting further ROS-genic diversion of electrons to O_2_. In addition, damping of the respiratory activity may cause accumulation of intermediates of the tricarboxylic acid cycle as well as of acetyl-CoA. When the latter accumulates it is known to cause lysine-acetylation and inhibition of function of a number of mitochondrial proteins including respiratory chain complexes [58,59]. The ensuing progressive mitochondrial damage/dysfunction is counteracted by upregulation of mitophagy. How and if these DCA-mediated mitochondrial alterations cause growth arrest and rewiring of the cell phenotype, in particular of the cancer stem cell compartment, remains to be established. However, a number of reported evidences indicate that a pro-oxidative state causes stem cell to exit from their undifferentiated state and induces/favors commitment [60,61]. Moreover, epigenetic modifications, such as those causing chromatin remodeling, regulate the balance between pluripotency and differentiation of stem cells [62,63]. Possibly the stalled TCA cycle may cause efflux of citrate in the cytosol where it releases acetyl-CoA, thus enhancing its availability for histone acetylation. Obviously, other uncharacterized DCA-targets may contribute or even dominate the observed drug effects. 

In conclusion, our results clearly indicate that the efficacy of DCA in inhibiting cancer cell growth is not always causally related to its documented stimulatory effect on the PDH activity and consequently reverse Warburg effect. Other off-targets must be mechanistically considered depending on the cell phenotype. In this context, the evidence, emerging from this study, that the CSC compartment in PDAC-derived cell lines might be affected by DCA treatment is relevant. It would be worthy to verify if this occurs in other cancer cell types and work is in progress in our lab in this direction. Recent research projects developing surface-functionalized, dichloroacetate-loaded nanoparticles [64], and multifunction drugs obtained by chemotherapeutic agents with DCA as ligand [65] could help to design the right targeted pharmacological formulation for developing new effective therapeutic strategies to fight pancreatic as well as other types of cancer.

## Supplementary Materials

The following available online at https://www.mdpi.com/2073-4409/8/5/478/s1: Figure S1: Effect of DCA on cell proliferation assessed by XCELLigence in medium containing low glucose; Figure S2: Scratch-healing; Figure S3: Effect of DCA on lactate production; Figure S4: Effect of 24 h DCA treatment on metabolic fluxes; Figure S5: Comparative effect of DCA on the metabolic fluxes in PDAC cell lines under condition of low glucose (LG) and high glucose (HG) culturing; Figure S6: Protein expression of factors involved in mitochondria fusion–fission (OPA1, MFN1/2); Figure S7: Assessment of stemness in PDAC cell lines.
