# Supplementary material for: Dichloroacetate Affects Mitochondrial Function and Stemness-Associated Properties in Pancreatic Cancer Cell Lines
Source: Cells. 2019 May 18;8(5):478. doi: 10.3390/cells8050478 (PMC6562462; doi:10.3390/cells8050478)
Supplement: Supplementary file 1 [file cells-08-00478-s001.pdf]

cells

MDPI

Article

Dichloroacetate Affects Mitochondrial Physiology And Stemness-Associated Properties In Pancreatic Cancer Cell Lines

Tiziana Tataranni[1], Francesca Agriesti[2], Consiglia Pacelli[3], Vitalba Ruggieri[4], Ilaria Laurenzana[5], Carmela Mazzoccoli[6], Gerardo Della Sala[7], Concetta Panebianco[8], Valerio Pazienza[9], Nazzareno Capitanio[10] and Claudia Piccoli*[11,12]

[1] Laboratory of Pre-Clinical and Translational Research, IRCCS-CROB, Referral Cancer Center of Basilicata, Rionero in Vulture (Pz), Italy; tiziana.tataranni@crob.it (T.T.); francesca.agriesti@crob.it (F.A.); vitalba.ruggieri@crob.it (V.R.); ilaria.laurenzana@crob.it (I.L.); carmela.mazzoccoli@crob.it (C.M.); gerardo.dellasala@crob.it (G.D.S.)

[2] Department of Clinical and Experimental Medicine, University of Foggia, Italy; consiglia.pacelli@unifg.it (C.P.); nazzareno.capitanio@unifg.it (N.C.)

[3] Division of Gastroenterology, IRCCS "Casa Sollievo della Sofferenza" Hospital, Italy; panebianco.c@gmail.com (C.P.); pazienza_valerio@yahoo.it (V.P.)

* Correspondence: claudia.piccoli@unifg.it; Tel.: 0881-588-060

Received: date; Accepted: date; Published: date

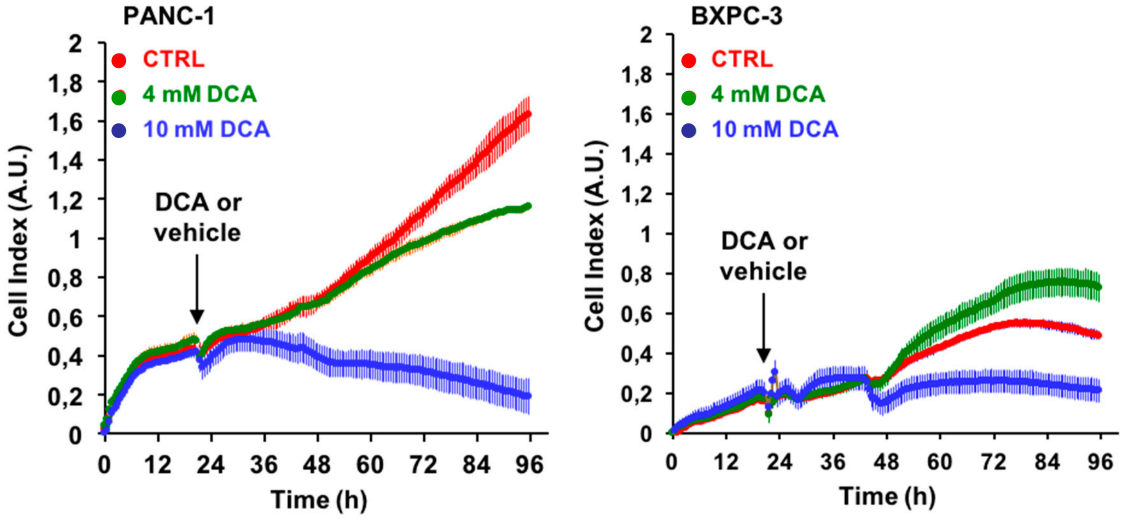

Figure S1. Effect of DCA on cell proliferation assessed by XCELLigence in medium containing low glucose. PANC-1 and BXPC-3 were seeded at the same density and cultured in RPMI supplemented with 1 mM glucose for 22 h before addition of 4 or 10 mM DCA or of vehicle (CTRL). The growth curve shown are representative of three independent time-courses. See Material and Methods of the main text for further details.

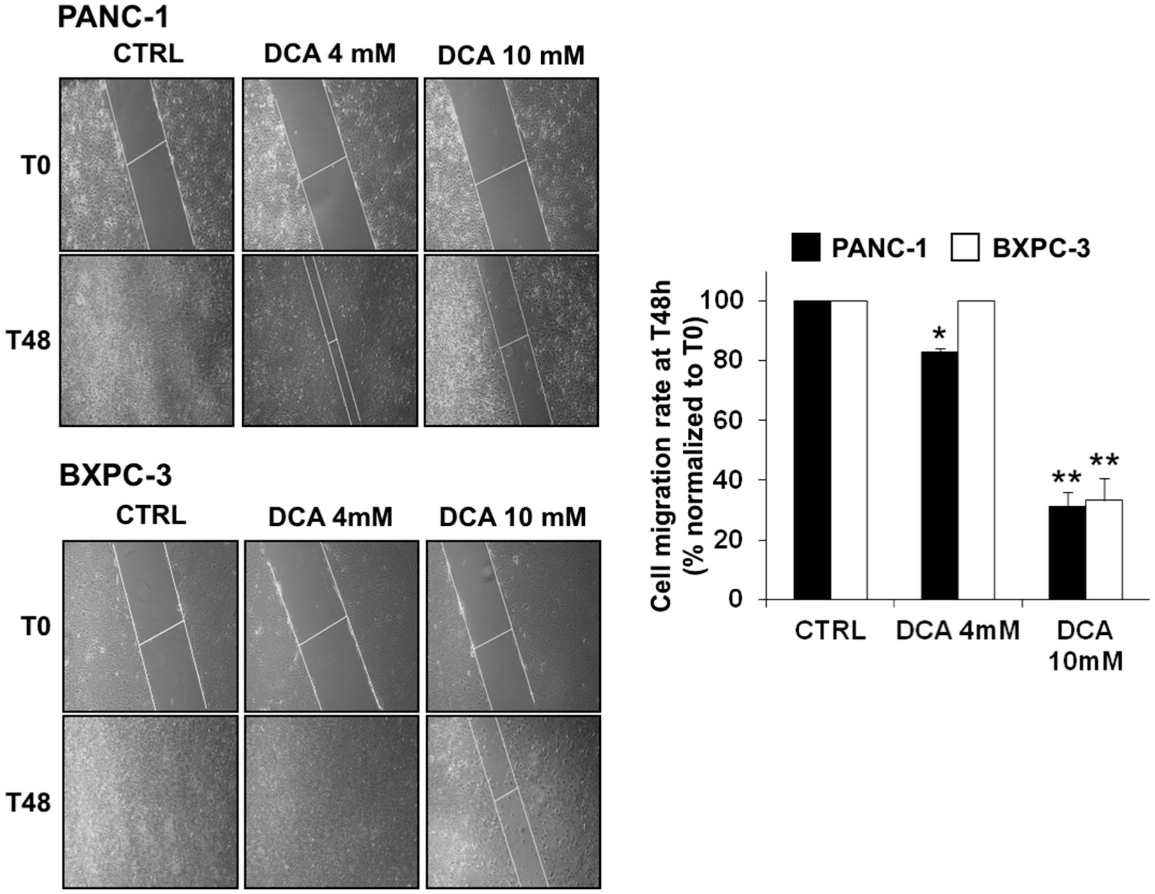

Figure S2. Scratch-healing assay. Representative scratch closure of PANC-1 (upper panel) and BXPC-3 (lower panel) observed under the microscope (50X) at T0 (before treatment) and after 48h of DCA 4 mM and 10 mM treatment (T48). Scratch assay was performed as described in Materials and Methods section. The histogram on the right show the cell migration rates of PANC-1 and BXPC-3 calculated by the width of scratch at T48h normalized to that at T0 per each condition and are expressed as mean ±SEM of three independent experiments. $^*p < 0.05$, $^{**}p < 0.01$ vs CTRL.

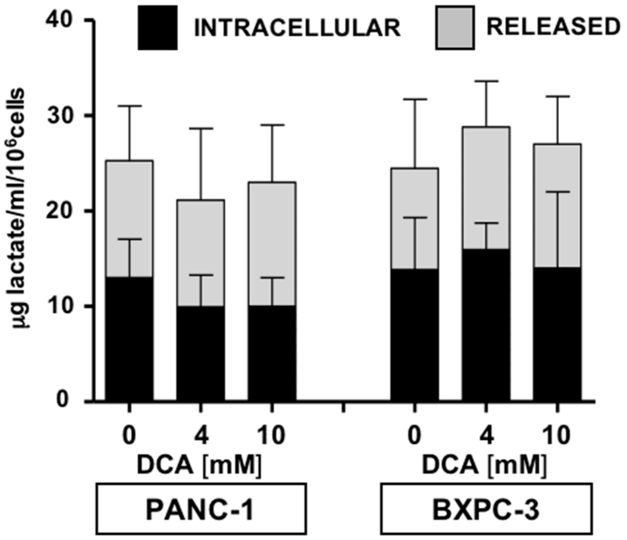

Figure S3. Effect of DCA on lactate production. Measurement of lactate in culture medium (grey column) or in culture cells (black column). 2x10^{6} cells were plated and treated with DCA 4mM and 10mM. After 24h of incubation lactate assay was performed as indicated in the Material and Method section.

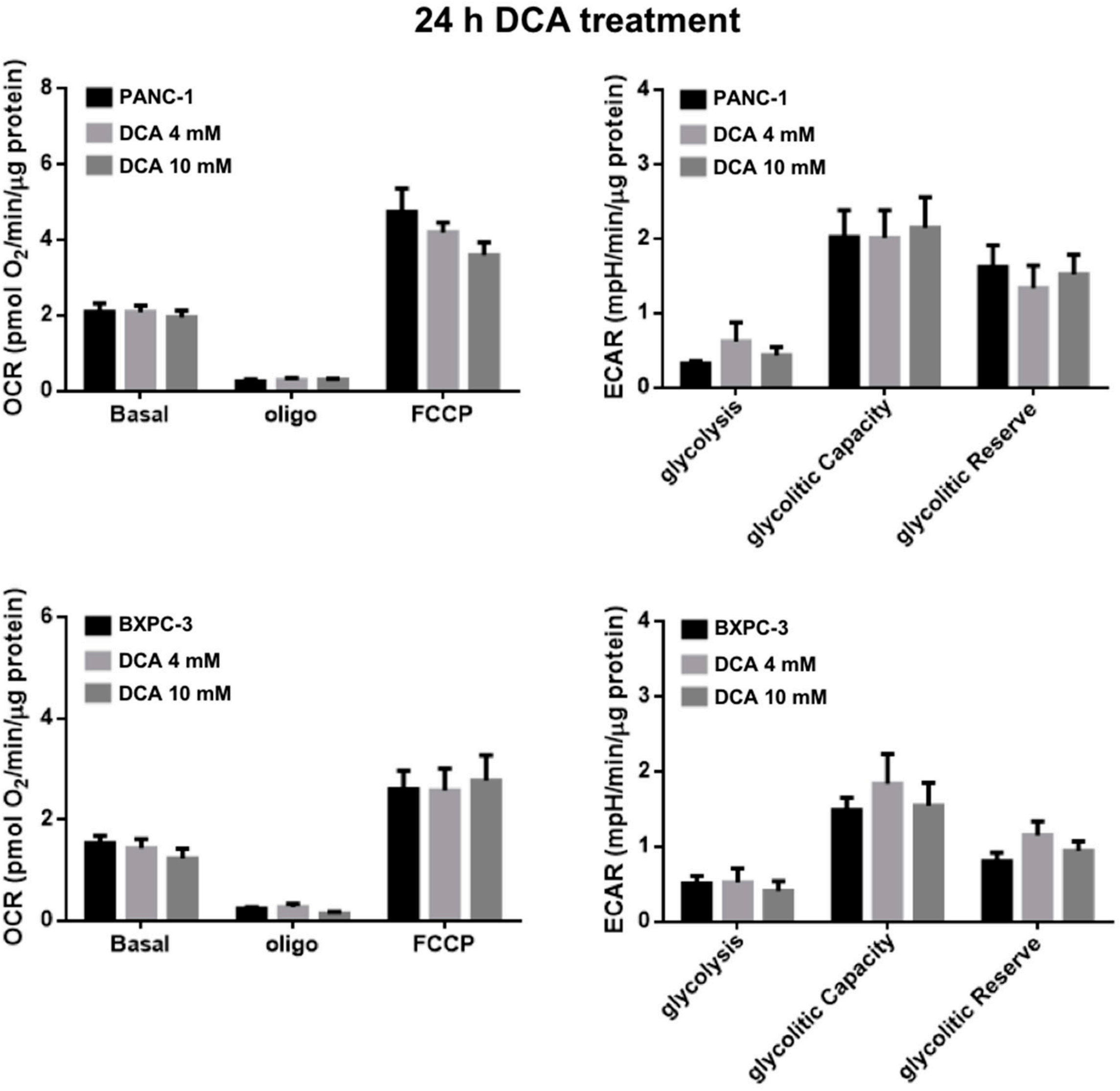

Figure S4. Effect of 24 h DCA treatment on metabolic fluxes. Oxygen consumption rate (OCR) performed by the SeaHorse platform in PANC-1 (Upper Panel) and BXPC-3 (Lower Panel); Extracellular acidification rate (ECAR) measured in PANC-1 (Upper Panel) and BXPC-3 (Lower Panel) treated with DCA 4mM and 10mM for 24h. Basal: resting OCR; Oligo: OCR measured after the addition of the ATP synthase inhibitor Oligomycin; FCCP: OCR measured after the uncoupler FCCP producing the maximal respiratory capacity. Glycolisis: resting ECAR; Glycolitic capacity: ECAR measure after the addition of olygomycin and FCCP and refers to maximal glycolytic activity with the Oxphos inhibited; Glycolytic Reserve: difference between ECAR measured in the presence of olygomycin+FCCP and under resting conditions. The bars represent the means ± SEM of 3 independent experiments carried out in 3 technical replicates under each condition.

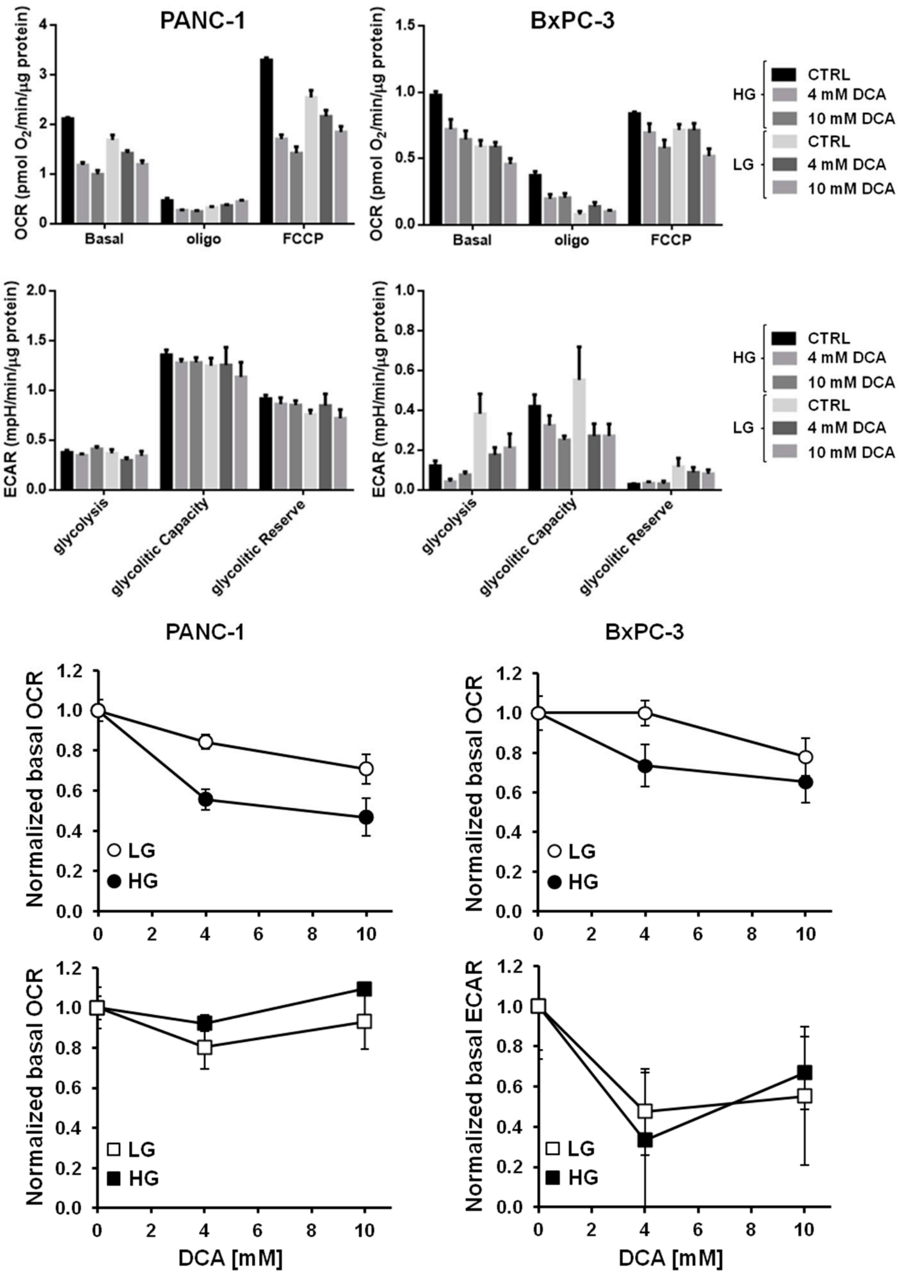

Figure S5. Comparative effect of DCA on the metabolic fluxes in PDAC cell lines under condition of low glucose (LG) and high glucose (HG) culturing. OCRs and ECARs were assessed in PANC-1 and BXPC-3 cell lines by the SeaHorse platform as previously described. LG and HG refer to 1 and 10 mM glucose in the RPMI medium. Incubation with the indicated concentrations of DCA was for 48 h before activities measurement. The upper histograms refer to the absolute OCRs and ECARs measured under basal condition or after the addition of oligomycin or the uncoupler FCCP and were all corrected for the residual activity in the presence of OCR or ECAR inhibitors. The lower graphs refer to basal OCRs and ECARs normalized normalized to their respective DCA-untreated controls. The values represent the means ± SEM of 3 independent experiments carried out in 3 technical replicates under each condition.

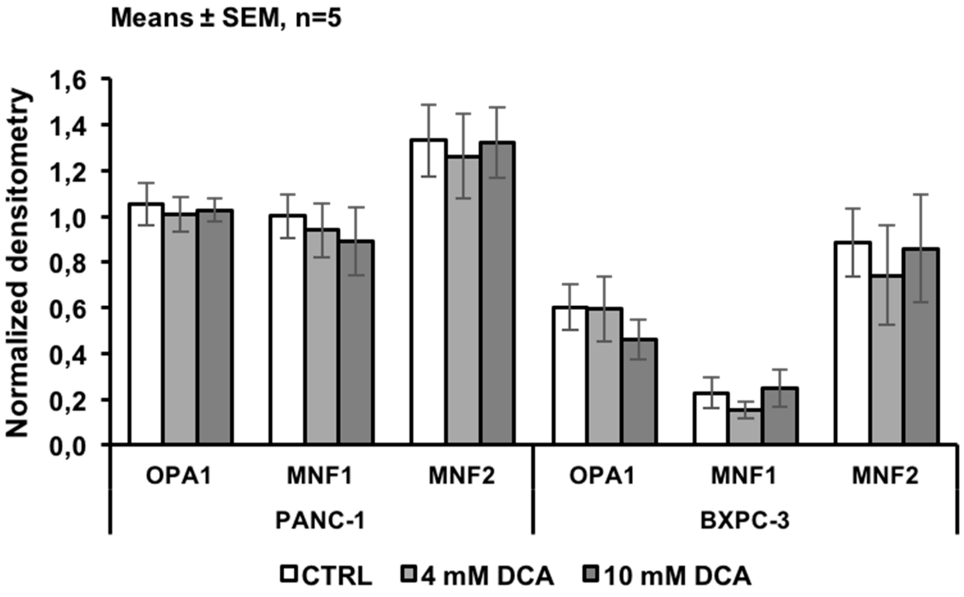

Figure S6. Protein expression of factors involved in mitochondria fusion (OPA1) – fission (MFN1/2). PANC-1 and BXPC3 line were incubated without or with the indicated concentrations of DCA for 48 h. After that cell protein extracts were subjected to immunoblotting as described in Materials and Methods of the main text. Densitometric analysis of the bands corresponding to the indicated proteins were normalized to that of β-actin. Bars are means ± SEM of three independent experiments.

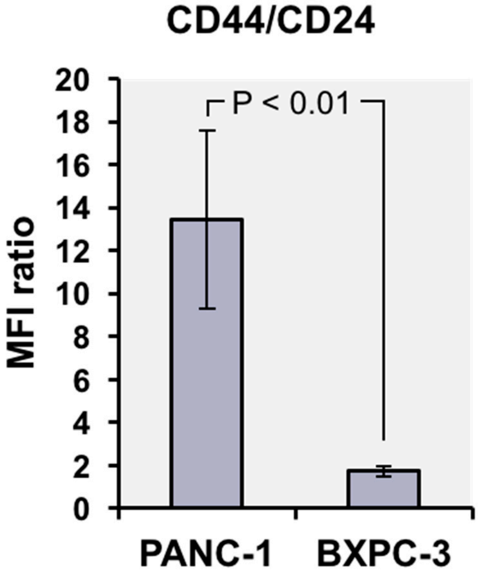

Figure S7. Assessment of stemness in PDAC cell lines. Expression of the surface markers CD44 and CD24 in PANC-1 and BXPC-3 cell lines was evaluated as mean fuorescence intensity by flow-cytometry as detailed in Materials and Methods of the main text. Bars are means ± SD of four independent experiments.
